# Density related effects on lifetime fecundity of *Heterakis gallinarum* in chickens

**DOI:** 10.1186/1756-3305-7-334

**Published:** 2014-07-17

**Authors:** Gürbüz Daş, Matthias Gauly

**Affiliations:** 1 University of Göttingen, Department of Animal Sciences, Albrecht-Thaer-Weg 3, 37075 Göttingen, Germany

**Keywords:** Cumulative egg excretion, Density-dependence, Inverse density-dependence, Lifetime fecundity, Nematode, Population dynamics, Worm length

## Abstract

**Background:**

Density related effects, both inverse density- and density-dependent, contribute to regulating population dynamics of parasites. We investigated whether density related effects are directly controlling lifetime fecundity of *Heterakis gallinarum*.

**Methods:**

Daily total numbers of *H. gallinarum* eggs in faeces samples (N = 1365) from chickens (N = 39) were quantified starting from 3 weeks (wk) post-infection (p.i.). The birds were necropsied 8 wk p.i., and intensity and demographic characteristics of infrapopulations were determined. Density related effects on cumulative egg excretion (CEE), lifetime fecundity and worm length were investigated with a segmented regression analysis.

**Results:**

For CEE, lifetime fecundity and female worm length, we determined highly similar parasite intensity thresholds (52–54 worms), which separated infrapopulations for influences of inverse density- and density dependence. CEE increased as parasite intensity increased up to an intensity of 52 worms. After this threshold, the relationship followed more of a horizontal line indicating impaired worm fecundity at higher parasite intensities. Lifetime fecundity was enhanced linearly in infrapopulations with up to 54 worms, but thereafter decreased gradually with increasing infrapopulation size. Female worm length increased linearly with elevating parasite intensity up to a threshold of 54 worms and thereafter declined with a rate of -0.014 mm for each additional worm. Lifetime fecundity and female worm length did not significantly differ between infrapopulations below and above the thresholds (P > 0.05). Lifetime fecundity was positively associated with the percentage of male worms (r = 0.44; P < 0.001), but negatively with absolute deviation from the theoretically expected sex-ratio in the infrapopulations (r = -0.56; P = 0.005). These relationships were stronger in infrapopulations below the threshold (r = 0.51 and -0.61, respectively), and were not significantly different from zero in the infrapopulations above the threshold (P > 0.05).

**Conclusions:**

Egg production of *H. gallinarum* is regulated by the effects of both inverse density- and density-dependent mechanisms, which result in similar average lifetime fecundity below or above intensity thresholds. In infrapopulations below the intensity thresholds, inverse density dependence effects on lifetime fecundity appear to result partly from sex-ratio fluctuations and impaired mating success of the nematode.

## Background

Host immune responses are considered as the primary mechanisms regulating population dynamics of nematodes within host [1,2]. In addition, density-dependent effects, which may partly be generated through immune responses, are known to contribute regulating parasite population dynamics [1,3,4]. For parasitic nematodes, the most pronounced density-dependent effects are mainly observed on establishment rate [5-7], worm length [2,8], fecundity [9,10] and sex-ratio [11]. In already established parasite infrapopulations, density-dependent effects may partly result in intraspecific interactions leading to increasing competition for finite resources e.g., food, space, mating among individuals as the size of infrapopulations become larger. As demonstrated by Bishop and Stear [9], density-dependent effects may not only cause a decrease in fecundity of individual worms, defined as faecal egg concentration per female worm, but may also conspicuously decrease total egg production of infrapopulations. Density-dependent effects apply in poultry nematodes, too. Permin *et al*. [5] observed that increasing infection doses of *Ascaridia galli* eggs resulted in almost the same number of worms per chicken, indicating a clear density-dependent effect on the establishment rate of the nematode. Establishment rate of the caecal worm, *Heterakis gallinarum*, has also been shown to be density dependent in a pheasant host-system [8]. Density related effects are, however, not limited to density dependence only, and thus are not always negative. The so called “Allee effect” refers to inverse density dependence at low densities [12]. Such a relationship has been demonstrated for the mean weight of female *Ascaris lumbricoides* which follows a pattern of initial facilitation followed by limitation with further increasing parasite density [13]. Similarly, *H. gallinarum* female worm length was shown to be influenced positively at low densities, whereas above a threshold it became density-dependent in heavily infected animals [8]. Although Thompkins and Hudson [8] considered the length of female *H. gallinarum* as a reliable indicator of fecundity, due to a high correlation with number of *in utero* embryonated eggs, inverse-density and/or density dependent effects have not so far been shown directly on worm fecundity. Therefore, this study aimed at investigating whether density related effects are regulating total egg excretion and fecundity of *H. gallinarum* in chickens. As the outcome of single faecal egg counts can suffer from methodological restrictions to some extent, we investigated density-related effects on long-term egg production outcomes including cumulative egg excretion of infrapopulations and lifetime fecundity of the nematode.

## Methods

### Chickens and *Heterakis gallinarum* infection

A total of 39 female white Leghorn (Lohmann Selected Leghorn) chicks were inoculated orally at the age of three weeks (wk) with approximately 200 embryonated eggs of *Heterakis gallinarum* that originated from *Histomonas meleagridis*-free batches. The preparation of the *H. meleagridis*-free infection material and the inoculation procedures have been described earlier [14]. The birds were purchased as one-day-old chicks and kept under group conditions for 40 days. Thereafter, they were placed into individual cages that provided the birds with free access to feed and water, and also allowed quantitative daily collection of individual faeces. No vaccination or anthelmintic treatment was applied.

### Nematode egg excretion and female worm fecundity

After a two-days adaptation period that ensured regular feed and water intake by the birds, daily faeces collections were started at the beginning of 4th wk post-infection (p.i). Faecal droppings excreted by each bird accumulated in a plastic bag-covered box underneath the cage. The total amount of faeces per bird/day was weighed, transferred into a plastic cup and stirred thoroughly for at least 3 minutes. A well-mixed sub-sample was derived from the daily amount of faeces of each bird. The number of eggs per gram of faeces (EPG) was quantified for the sub-sample using a modified McMaster counting technique with a minimum detection limit of 50 EPG and saturated NaCl as the flotation liquid (density ≥ 1.2 g/ml). A total of 1365 fecal samples were analyzed for EPG. Total number of eggs excreted per infrapopulation through faeces of the host-bird within 24 h (eggs per day, EPD) was then calculated daily for a period of 35 d. All the birds were necropsied 8 wk p.i. at an age of 11 wk to determine worm burdens and worm gender as described earlier [14,15]. Gender specific average worm length was determined by measuring all intact female and male worms of each bird. At necropsies, caecum size was also determined by measuring caecal lengths and full and empty (washed) weights.

### Definitions and statistical analyses

Daily *per capita* fecundity was calculated as EPD per female worm (EPD/fem). Cumulative egg excretion (CEE) by each nematode infrapopulation was estimated as the sum of total daily egg excretions (EPD) during the 35-days collection period. Average number of eggs excreted per female worm throughout the 35-d period was defined as lifetime fecundity (CEE/fem). Cumulative egg excretion and fecundity parameters were logarithmically (natural) transformed by using a function in the form of [Ln (*y*)] to correct for heterogeneity of variance and to produce approximately normally distributed data.

Daily fecundity (EPD/female) data were analyzed using ln-transformed [Ln (*y + 1*)] data with the repeated measures analysis of variance using Proc MIXED of SAS [16]. The statistical model included fixed effects of days post-infection (22–56) on which faeces samples were collected. As egg excretions of infrapopulations were measured over a period of 35 days, effect of repeatedly sampled host animal (subject) was included in the model as random. Daily fecundity was assumed to be correlated across measurements, and thus the covariance structure was set to be compound symmetry.

A segmented regression analysis [17] was used to investigate responses in the outcomes of infrapopulations (cumulative egg excretion, lifetime fecundity and worm length) to increasing parasite intensity.

The segmented regression included two conditional models:

$$\begin{array}{l} \begin{array}{cc} y_{i}=a+bx_{i} & \mathrm{for}\;x_{i}\leq x_{0} \end{array}\\ \begin{array}{cc} y_{i}=\left(a‐cx_{0}\right)+\left(b+c\right)x_{i} & \mathrm{for}\;x_{i}>x_{0} \end{array} \end{array}$$

which join at the knot *x*_0_ (the expected value *E* (y_*i*_ | *x*_0_) that is the same for both functions); *y*_*i*_ is dependent variable (e.g., lifetime fecundity); *x*_*i*_ is independent explanatory variable (parasite intensity); *a*, *b* and *c* are the parameters to be estimated. The parameters *a*, *b* and *c* can be considered as a constant (*a*) and slopes of the first (*b*) and the second (*c*) regression lines, respectively. The breakpoint (*x*_0_) is considered as a measure of threshold at parasite intensity after which outcomes of the response variables behave differently. The model was implemented in a NLIN procedure of SAS. To assess model adequacy, the error mean square (EMS) and a pseudo-goodness of fit (pseudo-R^2^) for non-linear regression were used. The pseudo-R^2^ [1- (SS error/SS corrected total)] was calculated manually as suggested by Schabenberger [18]. Graphical representation of data was carried out with SigmaPlot (V11).

### Ethical consideration

The infection dose (200 eggs) given to each bird was within the range of the worm burdens that can be observed in natural sub-clinical infections. All the experimental procedures followed the animal welfare rules. Data presented in this study were obtained from an approved project by the Lower Saxony State Office for Consumer Protection and Food Safety.

## Results

### Egg production and further characteristics of infrapopulations

**Figure 1:**
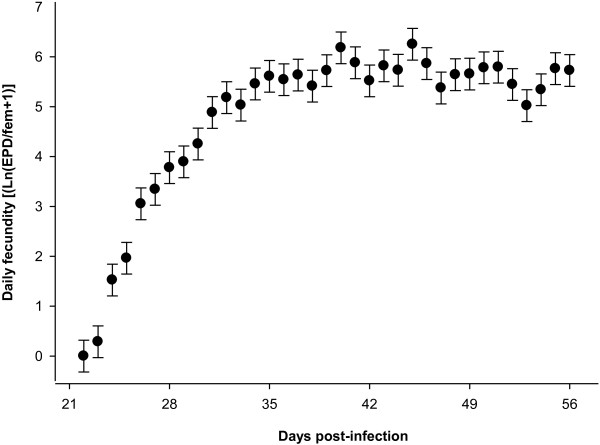
**Average daily fecundity (ln) in *Heterakis gallinarum *in experimentally infected chickens (N = 39).** Estimates (●) are least squares means and their SE on the error bars (Sample size, N = 1365).

On the first sampling day i.e. on day 22 post-infection (d.p.i), there was no egg positive sample. Average pre-patent period was 26 days (SD = 4.9). As shown in Figure 1, the average daily egg production per female worm (daily fecundity) increased sharply within the first two weeks of the patent period and reached a stable level that remained relatively constant for the rest of the study period. Average cumulative egg excretion per infrapopulation was roughly half a million eggs, which corresponded to an overall average of 416 eggs (SD =109) per female worm and day (Table 1). The experimental infection produced patent infections in all the birds. All the worms were mature i.e., there were no larval stages at 8 weeks p.i. On average, every third inoculated egg was recovered as mature worm, corresponding to an establishment rate of 33.5% and resulting in an average infection intensity of 67 (SD =53.7) worms per bird. Almost equal average numbers of female and male worms per chicken were harvested while overall average sex ratio was in favor of female worms (54.8%). Female worms were longer than males by 16%.

**Table 1:** **Cumulative egg excretion (CEE), overall average daily fecundity, worm counts and worm length in *Heterakis gallinarum *** **infrapopulations in experimentally infected chickens**^**1**^

**Item**	**Means ± SD**	**Min.**^**2**^	**Max.**^**2**^
CEE (in mi.)	0.51 ± 0.39	0.17	1.26
Av. daily fecundity^3^	416 ± 109	174	739
Female worms, #/bird	34 ± 25.8	2	100
Male worms, #/bird	33 ± 28.5	1	114
Total worm burden, #/bird	67 ± 53.7	3	214
Sex ratio, %^4^	54.8 ± 10.6	41.1	83.3
Female length, mm	10.7 ± 0.40	9.75	11.44
Male length, mm	8.97 ± 0.32	8.20	9.67

^1^The birds (N = 39) were infected with 200 *H. gallinarum*-eggs, and necropsied 8 weeks p.i.

^2^For worm length it is the lowest or the highest average of an infrapopulation, not a single worm length.

^3^Average daily number of eggs per female worm (CEE/fem/35d).

^4^Defined as [Sex ratio = (females/(females + males) × 100].

### Density related effects

**Figure 2:**
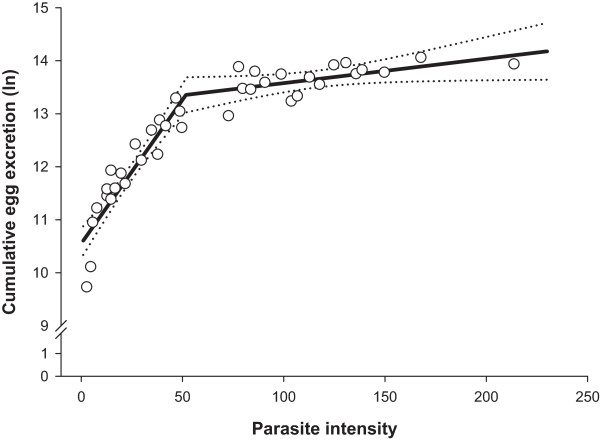
**Cumulative egg excretion (ln) in *Heterakis gallinarum *infrapopulations in relation to parasite intensity.** Open circles (*○*) are observed values, solid line (▬) is the predicted curve by segmented regression and the dashed lines (•••) indicate upper and lower 95% confidence bounds for the mean prediction.

**Figure 3:**
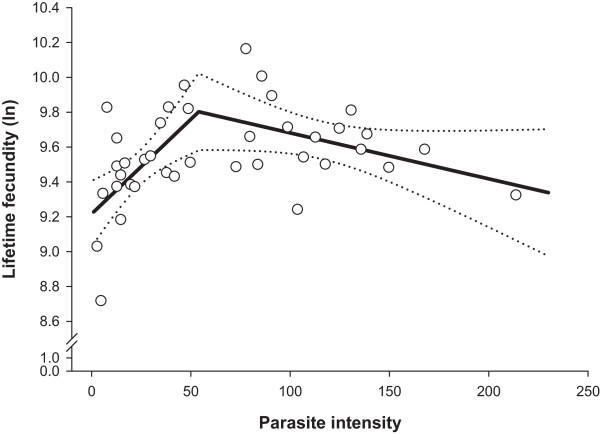
**Lifetime fecundity (ln) in *Heterakis gallinarum *infrapopulations in relation to parasite intensity.** Open circles (*○*) are observed values, solid line (▬) is the predicted curve by segmented regression and the dashed lines (•••) indicate upper and lower 95% confidence bounds for the mean prediction.

**Figure 4:**
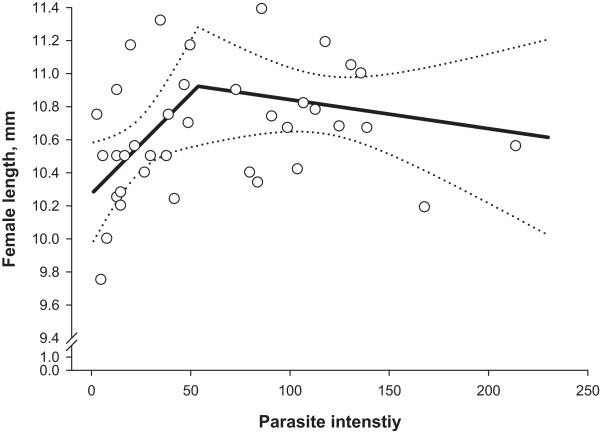
**Average female worm length in *Heterakis gallinarum *infrapopulations in relation to parasite intensity.** Open circles (*○*) are observed values, solid line (▬) is the predicted curve by segmented regression and the dashed lines (•••) indicate upper and lower 95% confidence bounds for the mean prediction.

The segmented regression models predicting non-linear relationships between parasite intensity (infrapopulation size) and cumulative egg excretion, lifetime fecundity or female worm length fitted significantly (Table 2; P ≤ 0.029). Goodnesses of model fit (pseudo-R^2^) ranged from 0.22 to 0.92 being the lowest and the highest for female worm length and for cumulative egg excretion (CEE), respectively. For all the predicted lines, joined with a breakpoint (*x*_0_) as a measure of threshold, the first slopes (*b*) were positive while the second (*c*) slopes were negative. The breakpoints for all the examined dependent variables were in a narrow range of parasite intensity of 52 to 54 worms (Table 2).As shown in Figure 2, CEE increased as parasite intensity increased up to a density of 52 worms per bird. Above this threshold, the relationship between CEE and worm burden followed more of a horizontal line. Figure 3 shows changes in lifetime fecundity as the response to increasing parasite intensity. Similar to CEE, lifetime fecundity enhanced linearly in birds infected with up to 54 worms, but thereafter decreased with increasing parasite intensity. Female worm length increased linearly with elevating parasite intensity up to a threshold of 54 worms and thereafter declined with a rate of -0.014 mm for each additional worm in the infrapopulations (Figure 4). There was no non-linear change in male worm length as a response to increasing worm counts (P = 0.463; data not shown).

**Table 2:** **Non-linear model parameters describing features of prediction lines (*a, b, c, x***_**0**_**) and model adequacy for cumulative egg excretion (CEE), lifetime fecundity (CEE/fem) and length of *Heterakis gallinarum *** **fitted to infrapopulation size by segmented regression analysis**

**Parameters**	**CEE (ln)**		**Lifetime fecundity (ln)**		**Female length**	
	**Estimate**	**SE**	**Estimate**	**SE**	**Estimate**	**SE**
*a*	10.55	0.137	9.22	0.093	10.27	0.152
*b*	0.054	0.005	0.011	0.003	0.012	0.005
*c*	-0.050	0.005	-0.014	0.004	-0.014	0.006
*x*_0_	52	4.49	54	11.41	54	18.04
P ≤	0.001		0.001		0.029	
EMS	0.110		0.051		0.135	
Pseudo-R^2^	0.92		0.35		0.22	

### Characteristics of infrapopulations below and above intensity thresholds

**Figure 5:**
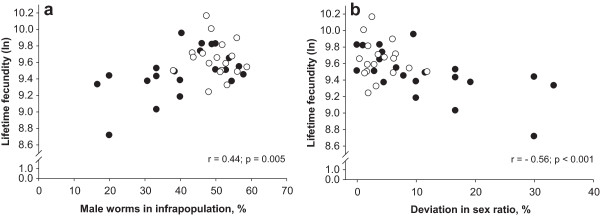
**Relationships between lifetime fecundity and percentage of male worms (a), and*s*ex independent absolute deviation from the theoretically expected sex-ratio (|sex ratio - 50%|) (b) in infrapopulations below (**●**) or above (**○**) the parasite intensity threshold (52 worms) determined by segmented regression (see Figures **2**, **3** and **4**).** Correlation coefficients (r) were calculated for pooled data (*● +* ○; N = 39).

*H. gallinarum* infrapopulations below and above the intensity threshold (52 worms) were compared with one-way-ANOVA to characterize outcomes and possible determinants in the infrapopulations. CEE was higher (P < 0.001) in the infrapopulations above the threshold than in those below. There were no significant (P > 0.05) differences in lifetime fecundity, female or male worm length between infrapopulations below and above the threshold. Similarly, there were no significant differences (P > 0.05) in caecum size (average caecum length, full or empty caeca weight) of birds harboring infrapopulations below or above the threshold. Sex composition of the infrapopulations was in favor (P < 0.001) of female worms at intensities below the thresholds (58.9%) than those above the thresholds (49.9%). Sex independent absolute deviation from the theoretically expected sex-ratio (|sex ratio in infrapopulation - 50%|) was higher (P < 0.001) at low parasite intensities (11.4%) than at high intensities (4.1%).In general, lifetime fecundity was positively associated with the percentage of male worms (Figure 5a; r = 0.44; p < 0.001), but negatively with absolute deviation from the theoretically expected sex-ratio in the infrapopulations (Figure 5b; r = -0.56; p = 0.005). These relationships were stronger in infrapopulations below the threshold (r = 0.51 and -0.61, respectively), and were not significantly (p > 0.05) different from zero in the infrapopulations above the threshold (r = -0.08 and r = -0.12, respectively). There was a moderate positive correlation (r = 0.48; p = 0.002) between lifetime fecundity and female worm length.

## Discussion

We investigated whether and to what extent density related effects, both inverse density and density-dependent, influence long-term egg production outcomes of *Heterakis gallinarum* infrapopulations in a chicken-host system. It was demonstrated for the first time for *H. gallinarum* that lifetime fecundity is influenced by both inverse density and density-dependent effects, separated by a density threshold of 52–54 worms in chickens. In addition, our results confirm fully the inverse density and density-dependence in female worm length that was previously demonstrated by Thompkins and Hudson [8]. Thompkins and Hudson [8] determined a higher threshold (96 worms) above which density dependent effects replaced inverse density dependency. The differences in the thresholds may not only be attributed to different host species, but also to some other factors e.g. age of birds, caecum size, age of infections and infection intensities. Co-existence of both inverse density and density dependency mechanisms regulating parasite population dynamics, particularly in nematodes, seems to be rare. Although there are at least two studies describing existence of both inverse density and density dependency in parasitic nematodes [8,13], these studies rely on evidence obtained from relationships between parasite intensity and female worm size, measured either as length [8] or weight [13]. As confirmed in the present study, female worm length, but not male worm length, is a valuable indicator of fecundity and responds to increasing parasite intensity in a parallel way as lifetime fecundity does. However, lifetime fecundity correlated linearly with female worm length only moderately (r = 0.48). In the existence of both inverse density and density dependent mechanisms on both lifetime fecundity and on female worm length, linear relationships between worm length and fecundity might have weakened. The model fit parameters of the segmented regression were better for lifetime fecundity than for female worm length. Since parasite survival and fecundity, but not parasite size, are the two parameters of importance in the generation of epidemiological patterns and the determination of evolutionary fitness of a parasite species, density related effects on parasite size must be translated into influences on parasite survival and fecundity [1]. Our results suggest that the effects on length translate into effects on fecundity. For *H. gallinarum*, female worm length, but not directly fecundity, had been shown earlier to be regulated by the density related effects. This was probably due to difficulties in quantification of egg excretion, which has often been problematic mainly because of the periodicity in the caecal faeces excretion of the birds. The caecal faeces, which contains *Heterakis* eggs, is passed to the external environment periodically [19], according to Klasing [20] only once or twice a day. That is why examining random regular faecal samples, which mostly originate from non-caeca-intestines, may result in false negative faecal egg counts (FEC) even though in heavily Heterakis-infected animals [21]. We have previously shown that analyzing well-mixed samples obtained from the daily total amount of faeces provides highly repeatable FEC that are also representative of *H. gallinarum* infrapopulation size, i.e. show high positive correlations with actual worm burdens [15].

Density dependent effects on fecundity have traditionally been assessed by plotting faecal egg concentration per female worm (EPG/female) against parasite intensity [2]. However, fecundity in the form of EPG/female seems to be a function of two imprecise estimates. Excluding technical limitations with egg counting methods [22], egg concentration in faeces (EPG) is easy to determine and it ensures contribution of all prolific females in an infrapopulation, although accuracy and precision of EPG are also influenced by a couple of faeces-related factors. The most pronounced ones are the daily amount and the water content of faeces, which may result in under- or overestimation of EPG [15,23]. On the other hand, accuracy of worm recoveries may not always be high, particularly in large hosts with predilection sites of high volumes. Additionally, plotting worm burden against its inverse multiplied by FEC may obscure the true biological relationships [2]. Thus, fecundity expressed as EPG/female is regarded as a rather imprecise estimate, and extreme cases may even be statistical artefacts [24]. This may particularly be true for estimates in the form of EPG/female or even to some extent for EPD/female relying on a single measurement. The most apparent feature of the data used for the present study was that the total egg excretion outcomes of the infrapopulations were on the basis of the sum of 35 individual daily total egg excretions. Thus, compared with measurement error of a fecundity estimate relying on a single faecal egg count (e.g., EPG/fem or EPD/fem), the measurement errors of the cumulative egg excretion estimates must have been much smaller. However, lifetime fecundity estimates do not rely only on the cumulative egg excretions, but also on the worm counts, which could be quantified only once and just at the end of the experimental period. Therefore, any change that might have taken place in the worm counts of the birds during the faeces collection period has the potential to influence accuracy of the lifetime fecundity estimates. However, this seems unlikely for three reasons. First, experimental infections with *H. gallinarum* result in linearly decreasing infection intensities up to 30 d.p.i., but thereafter, once patency is achieved, remain almost constant [8]. This implies stability of the patent infections with mature worms descending from a single inoculation. Second, as shown with the daily fecundity estimates (Figure 1) *per capita* egg production of the nematode was highly stable starting from the 5th wk. p.i. This also supports the stability when patency is achieved. Third, as reinfections were prevented in the experimentally-infected birds through daily removal of faeces, the number of egg-producing female worms is unlikely to have increased over the course of the study. Thus it can be inferred that total egg excretion and lifetime fecundity data shown in this study resulted from stable patent-infrapopulations. Population stability is indeed a characteristic feature of helminth infections [25]. It may therefore be hypothesized that cumulative egg excretion is not merely an outcome of certain number of female worms within a given time, but it may also reflect follow-up regulatory effects of the host immune system which initially have to act on establishment of larvae [8] and eventually contribute to determining number of egg producing females as well as their fecundity. The experimental infection produced worm burdens that were comparable to the levels observed both in naturally occurring [21,26,27] and in experimentally performed *H. gallinarum* infections of gallinaceous birds [8,14,23,28,29].

In contrast to natural infections, experimental infections may provide the possibility of investigating the effects of different mechanisms regulating parasite population dynamics separately. For *H. gallinarum*, regulatory mechanisms not only involve host-immune responses and density related mechanisms, which may also be interlinked with host immune responses [1,3,4], but also presence of another (hyper-)parasite, *Histomonas meleagridis*[30,31], which is vectored directly by *H. gallinarum* at some cost. We previously showed that concomitant infections of chickens with *H. meleagridis* and *H. gallinarum* result in reduced number of *H. gallinarum,* whose growth is also impaired, indicating a direct regulatory mechanism on both establishment and growth, and indirectly implying an impaired fecundity due to the stunted growth [14]. For the present study, however, we eliminated *H. meleagridis* so that it was excluded from the driving forces regulating population dynamics of *H. gallinarum*. Therefore, in this study regulatory effects on population dynamics of *H. gallinarum* were mainly restricted to host immune responses and density related effects. However, these effects cannot be separated in experimental infections either. Thus, next to the density related effects, the realized cumulative egg production and lifetime fecundity might have been influenced by the follow-up effects of the immune system. It may be assumed that the host-immune system might have operated better on larval establishment in birds harboring infrapopulations below the thresholds. Existence of infrapopulations under influence of either inverse density or of density dependent-effects may provide insight for a better understanding of host immune responses. After having determined highly similar thresholds in parasite intensity (52–54 worms) at which cumulative egg excretion, lifetime fecundity and female worm length started to respond increasing parasite intensity differently, we further investigated relationships between cumulative egg excretion and caecum size as well as sex ratio, to test whether density related effects could partly be explained by intraspecific competition for space and/or mating. Density dependence in parasite fecundity may result from both intraspecific competition and generation of immune responses, which increase disproportionally in efficiency as antigenic stimulation increases with increasing parasite intensity [1,3]. Because there were no significant differences in the size of predilection site of the infrapopulations below or above the parasite threshold intensities, it seems less likely that density-dependent effects resulted merely from the intraspecific interactions, but high probably co-influenced by the regulatory host immune responses as known for other nematodes [3]. Average lifetime fecundity as well as female worm length did not differ between infrapopulations below or above the thresholds. This may indicate similar efficacy of inverse density- and density-dependency mechanisms in regulation of fecundity.

Inverse density dependence may result from sex-ratio fluctuations and impaired mating success of the sexually reproducing nematode. The positive correlation between lifetime fecundity and percentage of male worms, particularly in infrapopulations below the threshold, as well as the negative correlation between lifetime fecundity and deviation from the theoretically expected sex-ratio, point to such a mechanism. The two parameters, male-ratio and deviation from the expected sex-ratio may theoretically, however, suffer from disproportional relative differences of numbers at low parasite intensities. Because small numbers have higher relative differences, a higher deviation may be expected in sex ratio at low parasite intensities, implying a high but sex-unbiased percentage of either female or male worms at low intensities. And correspondingly, as infrapopulation size increases, percentages of male and female worms must approach the theoretically expected ratio (50%) as predicted by the central limit theorem. However, it is worthy of notice that deviations at low intensities were not sex-independent and the infrapopulations were composed more of females. This may indicate density dependence in sex-ratio in favour of female worms at low intensities, which is known for many other dioecious helminth parasites [11,32,33]. With sex-manipulated infection models, Jungersen *et al.*[34] showed that egg production in *Ascaris suum*, a parasitic nematode of pigs sharing the same family (Ascarididae) with *H. gallinarum*, is dependent on frequent matings, indicating a crucial role of males on fecundity. Because there was no difference in caecal length between infrapopulations below and above the thresholds, it may be assumed that lower parasite intensity in a relatively larger space might have negatively influenced mating success of female worms at low parasite intensities, particularly where male worms were additionally less abundant. With experimentally conducted intercaecal migration studies, Fine [35] demonstrated that both female and male *H. gallinarum* were capable of migrating between caeca in either direction, and the migration appeared to be related to sexual activity of worms. However, these observations were made 14–17 days after worm transfer into caeca, thus it remains unknown to what extent worm mobility compensates negative effects of deviations from the theoretically expected sex ratio on worm fecundity at low parasite intensities.

## Conclusions

Egg production of *H. gallinarum* is regulated by the effects of both inverse density and density-dependent mechanisms which results in similar average lifetime fecundity below or above worm intensity thresholds. In infrapopulations below the intensity threshold, inverse density-dependent effects on lifetime fecundity appear to result partly from sex-ratio fluctuations and impaired mating success of the nematode.

## Competing interests

The authors declare that they have no competing interests.

## Authors’ contributions

GD and MG conceived the study. GD performed the experimental work, analyzed and interpreted the data, prepared a draft manuscript. MG reviewed the draft manuscript. MG provided funding. Both authors read and approved the final version of the manuscript.
